# Amalgamation?

**Published:** 1905-11

**Authors:** 


### Amalgamation?

THE New York State Medical Association, at its annual meeting held October 17-20, 1905, voted on the question of union with the Medical Society of the State of New York. Dr. Wisner R. Townsend offered the resolution for amalgamation, which was seconded by Dr. E. Eliot Harris. The vote as recorded stood: for, 1,517; against, 2; not voting, 295. It is reported that the negative votes were cast by,—we cheerfully give their names,—Glover Arnold, of New York, and Thomas D. Strong, of Westfield. Unless the unforseen happens it would seem as if the expectations were justifiable that at the centenniary meeting of the state society next winter the union of the two state medical bodies will become an accomplished fact. Let us hope and pray.

